# Complete genome sequence of lytic bacteriophage BAU.Micro_SLP-22 infecting avian *Salmonella* spp.

**DOI:** 10.1128/mra.00894-24

**Published:** 2024-12-19

**Authors:** Md. Arefin Kallol, Mohammad Ferdousur Rahman Khan, Jahangir Alam, Md. Bahanur Rahman, Marzia Rahman

**Affiliations:** 1 Department of Microbiology and Hygiene, Faculty of Veterinary Science, Bangladesh Agricultural University, Mymensingh, Bangladesh; 2 National Institute of Biotechnology, Savar, Dhaka, Bangladesh; Katholieke Universiteit Leuven, Leuven, Belgium

**Keywords:** bio-controlling agent, *Salmonella *phage, drain water, lytic bacteriophage

## Abstract

A lytic bacteriophage, BAU.Micro_SLP-22, was isolated from drain water in search of bio-controlling agents against avian salmonellosis. The phage genome is comprised of 59,738 bp with 56.96% guanine–cytosine content, encoding 81 protein-coding genes containing no transfer RNAs, antibiotic resistance, virulence, temperate marker, and clustered regularly interspaced short palindromic repeat coding sequences.

## ANNOUNCEMENT

Finding effective stratagems that serve as alternatives to antibiotics or functioning along with the antibiotics are consequently essential due to the multi-drug-resistant (MDR) bacterial epidemics in recent years (1). Application of bacteriophages has drawn attention to treat MDR bacterial infections effectively (2).

We report, here, a lytic bacteriophage, BAU.Micro_SLP-22, isolated from drain water in Mymensingh, Bangladesh (24°43′17.3″N 90°26′20.6″E), as a potential bio-controlling agent against avian salmonellosis. MDR avian *Salmonella enterica* strain MBR-MFRK-23, (characterized by [3]) was used as a bacterial host. Sample drain-water was co-cultured with host bacteria in 10× Nutrient Broth after centrifugation (12,000 *g*, 10 min) and filtration (0.22 µm). Then, phage isolation, purification (five rounds of plaque purification), and higher titer lysate preparation (10^11^ PFU/mL) were performed following standard protocols from (4). Genomic DNA was extracted by phenol–chloroform–isoamyl alcohol/sodium dodecyl sulfate DNA extraction method according to Actinobacteriophage database protocol resources (https://phagesdb.org). DNA library was prepared using Illumina TruSeq Nano DNA Library Prep kit (paired-end reads with an average length of 150 nucleotides) and sequenced with Illumina NovaSeq 6000 sequencing platform. Sequence read quality was evaluated using FastQC (Galaxy Version 0.74) (5) and trimmed with trimmomatic (Galaxy Version 0.38.1) (6). The phage genome was assembled using SPAdes (Galaxy Version 3.15.4) (7) considering the following: Operation mode - only-assembler, Pipeline options- Careful, k-mer detection option- Auto. Phage genome termini were determined using PhageTerm (Galaxy Version 1.0.12) (8). Genome assembly quality was assessed with Quast (Galaxy Version 5.2.0) (9), and genome completeness was determined with CheckV version 0.8.1 from PhageScope (10). Genome annotation was performed using Pharokka (Galaxy Version 1.3.2) (11). Taxonomy of the phage was determined based on phylogenetic lineages (BLASTn) from closely related genomes in the GenBank database (12). For all software, default parameters were applied except where otherwise mentioned.

The complete genome of phage BAU.Micro_SLP-22 encompasses 59,738 bp with 56.96% guanine–cytosine content, 81 putative protein-coding sequences (33 coding sequences [CDS] encode known functional proteins and 48 CDS encode hypothetical proteins) with 256× genome coverage, zero transfer RNAs, no clustered regularly interspaced short palindromic repeats, and with direct terminal repeats (DTR) genome termini. Analysis with PhageLeads (13) reveals that there is no presence of virulent factor, antimicrobial resistance, or temperate lifecycle encoding genes in the genome of the phage BAU.Micro_SLP-22.

Bacteriophage BAU.Micro_SLP-22 is a dsDNA virus, and according to the National Center for Biotechnology Information (NCBI) taxonomy database, the phage belongs to a species of *Jacunavirus* genus in the family of *Casjensviridae* (NCBI: txid3064292). Blastp (NCBI) analysis of phage BAU.Micro_SLP-22 major head protein (WPK30458.1) demonstrated 69.41% to 69.97% similarities (98% Query coverage) with the major head protein of Salmonella phages SPN19, FSL SP-030, and YSD1 (accession nos. YP_006990304.1, YP_008239847.1, and YP_009833586.1, respectively), whereas the phage BAU.Micro_SLP-22 terminase large subunit protein (WPK30463.1) exhibited 67.15% to 67.58% (Query coverage 99%–100%) similarities with the terminase large subunit protein of Salmonella phages FSL SP-088, SB5, vB_SentM_sal3, and Chi (accession nos. YP_008239960.1, WNO29180.1, QMV34379.1, and YP_008058169.1 sequentially).

**Fig 1:**
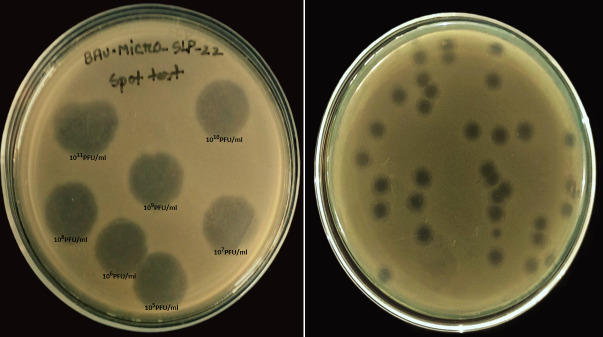
(Left) Lytic zone on host bacterial lawn by bacteriophage BAU.Micro_SLP-22 (spot test assay). (Right) Plaques formed by phage BAU.Micro_SLP-22 on a double-layer agar plate with host bacteria after 12 h of incubation at 37°C (plaque assay).

Strong lytic characteristic and plaque morphology of the phage BAU.Micro_SLP-22 are shown in Fig. 1. Finally as a short closing remark, bacteriophage BAU.Micro_SLP-22 would be a potential bio-controlling agent against avian salmonellosis.

## Data Availability

The complete genome sequence data of phage BAU.Micro_SLP-22 are available in GenBank, NCBI, archived as GenBank accession no. OR699283.2, Sequence Read Archive (SRA) accession no. SRR25434661, and BioProject accession no. PRJNA998627.
